# Yunnan Baiyao reduces hospital-acquired pressure ulcers via suppressing virulence gene expression and biofilm formation of *Staphylococcus aureus*

**DOI:** 10.7150/ijms.33723

**Published:** 2019-07-21

**Authors:** Jun Liu, Mufa Cai, Huimin Yan, Jiawu Fu, Guocai Wu, Zuguo Zhao, Yi Zhao, Yan Wang, Yuanming Sun, Yongke You, Liyao Lin, Juan Huang, Riming Huang, Jincheng Zeng

**Affiliations:** 1 Laboratory of Pathogenic Biology, Guangdong Medical University, Zhanjiang 524023, China;; 2 Dongguan Key Laboratory of Medical Bioactive Molecular Developmental and Translational Research, Guangdong Provincial Key Laboratory of Medical Molecular Diagnostics, Guangdong Medical University, Dongguan 523808, China;; 3 Department of Clinical Laboratory, Affiliated Hospital of Guangdong Medical University, Zhanjiang, Guangdong 524001, China;; 4 Department of Neurology, Affiliated Hospital of Guangdong Medical University, Zhanjiang 524001, China.; 5 Department of Blood Internal Medicine, Affiliated Hospital of Guangdong Medical University, Zhanjiang 524001, China;; 6 Guangdong Provincial Key Laboratory of Food Quality and Safety, College of Food Science, South China Agricultural University, Guangzhou 510642, China;; 7 School of Chinese Medicine, The University of Hongkong, Pokfulam, Hongkong;; 8 Department of Cardiothoracic Surgery, Affiliated Hospital of Guangdong Medical University, Zhanjiang, Guangdong 524001, China.

**Keywords:** Yannan Baiyao, hospital-acquired pressure ulcers, * Staphylococcus aureus*, * agr* system, virulence factors, biofilms

## Abstract

Yunnan Baiyao (YB) as a kind of famous Chinese herbal medicine, possessed hemostatic, invigorating the circulation of blood, and anti-inflammatory effects. Identifying strategies to protect patients at risk for hospital-acquired pressure ulcers (HAPU) is essential. Herein, our results showed that YB treatment can effectively reduce the acne wound area and improve efficacy in a comparative study of 60 cases HAPU patients with *S. aureus* positive of acne wound pathogens. Furthermore, YB inhibited HIa expression and suppressed accessory gene regulator (*agr*) system controlled by regulatory RNA II and RNA III molecule using pALC1740, pALC1742 and pALC1743 *S*. *aureus* strain linked to gfp_uvr_ reporter gene. Moreover, YB downregulated *cao* mRNA expression and inhibited coagulase activity by RT-PCR*,* slide and tube coagulase test. Additionally, YB downregulated *seb*, *sec*, *sed*, and *tsst*-1 mRNA expression to suppress enterotoxin and tsst-1 secretion and adhesion function related genes* sarA*, *icaA*, and* cidA* mRNA expression. Taken together, the data suggest that YB may reduce HAPU via suppressing virulence gene expression and biofilm formation of *S. aureus.*

## Introduction

Hospital-acquired pressure ulcers (HAPUs)-induced skin and soft-tissue injuries are the most common problems encountered in hospitalized patients and those in long-term institutional care and threat to patients health 1. HAPUs may prolong the hospital stay and lead to increased medical costs. The Healthcare Cost and Utilization Project (HCUP) report estimated that the average cost of treating pressure injuries is $37,800 per patient 2. Additionally, the main infectious complications that can develop from HAPUs are cellulitis, abscess, osteomyelitis, and bacteremia 3. Therefore, reduction and prevention of pressure ulcers is one of the greatest healthcare challenges to reducing patient harm.

*Staphylococcus aureus* (*S. aureus*)*,* known as one of the most frequent strain usually causes food poisoning and widespread infection, and is a risk factor for exacerbating HAPUs. It comes from superficial skin and other soft tissue infections to life threatening toxic shock, skeletal system, circulatory system, respiratory system, implantable medical devices, and the blood stream 4-6. At present, *S. aureus*-associated HAPUs have been increasing year by year. However, due to the abuse of antibiotics, *S. aureus* is seriously resistant to drugs and the therapeutic effect of HAPUs is limited 7. Therefore, there is an urgent need for a novel strategy that does not cause microbial resistance.

In Asia, traditional Chinese medicine for treatment of infection has a long history 8. Yunnan Baiyao (YB) as a kind of famous Chinese herbal medicine, possessed hemostatic, invigorating the circulation of blood, and anti-inflammatory effects 9, 10. It has been used for more than 100 years and has not caused any infection, indicating that YB may be a promising drug-resistant drug.

Our previous study proposed for the first time that sub-MIC value of the aqueous extract of YB could efficiently inhibit secretion of toxins, movement of flagellum and pili, and the formation of biofilms of gram-negative bacterium* Pseudomonas aeruginosa* 11*.* This prompted us to pay considerable attention to understand whether YB has its potential influence on *S*. *aureus* and HAPUs patients. This study was designed to investigate the effect of YB on HAPUs patients and sub-MICs of YB active ingredients on the expression of *S*. *aureus* agr system, virulence factors and biofilms. Combined with our previous reported, herein, we found that YB treatment can effectively reduce the acne wound area and improve efficacy in a comparative study of 60 cases HAPU patients with *S. aureus* positive of acne wound pathogens. Furthermore, antibacterial effect of YB on *S. aureus* also showed that YB may reduce HAPU via suppressing virulence gene expression and biofilm formation of *S. aureus.*

## Materials and methods

### Patients

60 cases of hospital-acquired pressure ulcers (HAPU) patients who were hospitalized at Affiliated Hospital of Guangdong Medical University were enrolled in this study. Subjects with missing at least one item from the following: admission method, consciousness status, pain, and Braden subscales were excluded. The demographic and clinical characteristics for all study subjects are described in Table 1. This study was approved by the Internal Review and the Ethics Boards of Guangdong Medical University. Informed written consent was obtained from all study subjects.

### Patient treatment with Yunnan Baiyao (YB)

The patient performed routine debridement of the wound to completely remove the necrotic tissue from the wound, and then cleaned the wound with hydrogen peroxide and sterile saline until the fluid that was discharged was clean. The wound surface is exposed and the appropriate infrared irradiation parameters are selected according to the area and location of the wound surface for 20~30 min, the distance is 30~50 cm, and the intensity is based on the patient's feeling of warmth. The treatment group was prepared into a paste by adding appropriate amount of Yunnan Baiyao (YB group Co, Ltd) according to the size of the wound surface, and then applied to the wound surface with a sterile cotton swab, covered with sterile Vaseline gauze and covered with sterile gauze. The control group was filled with Vaseline oil yarn to fill the pressure wound, and then covered with sterile gauze, and changed once a day. The effects of the two groups were evaluated after 20 days of treatment, and divided into four categories according to the treatment effect. (1) Effective: wound healing, scarring and shedding. (2) Markedly effective: no secretions, shrinkage of the wound, granulation tissue growth. (3) Improvement: the exudate is reduced and the wound is not enlarged. (4) Ineffective: wound does not heal, there is still exudate.

### Bacteria strains

*S*. *aureus* strain pALC1740 (*hla* promoter fused to a gfp_uvr_ reporter gene), pALC1742 (containing an RNAII promoter linked to gfp_uvr_ reporter gene) and pALC1743 (containing an RNAIII promoter linked to gfp_uvr_ reporter gene) were a kind gifted by Professor Ambrose L. Cheung at Dartmouth College.* coa*^+^ *S*. *aureus*, *icaA*^+^ *S*. *aureus*, *sarA*^+^ *S*. *aureus*, *cidA*^+^ *S*. *aureus*, *sea*^+^ *S*. *aureus*, *seb*^+^ *S*. *aureus*, *sec*^+^* S*. *aureus*, *sed*^+^ *S*. *aureus*, *see*^+^ *S*. *aureus* and *tsst-1*^+^ *S. aureus* strains were collected from Affiliated Hospital of Guangdong Medical University, from April 2013 to February 2017.* S*. *aureus* ATCC29213 was purchased from National Institutes for Food and Drug Control (China).

### Preparation of drug extract

According to the reported method 12, 100 g YB powder purchase from YB group Co, Ltd. was extracted with 500 mL ultrapure water at 50 ^o^C for 24 h. The aqueous extract was centrifuged twice at 25000 rpm for 60 min. The supernatant liquor of the extract was concentrated in vacuo to 100 mL aqueous extract, then lyophilized (YO0230, Thermo). The lyophilized powder was stored at -50 ^o^C before it was used.

### Determination of MIC and sub-MIC

Based on the reported method 13, the MIC and sub-MIC of active components in YB were determined by tube dilution method. The MIC value of YB for *S. aureus* was determined by two-old macro-dilutions in Mueller-Hinton broth with an inoculum of 5×10^5^ colony forming unit (CFU)/mL. The final concentration of active components in YB was 512 mg/mL to 0.125 mg/mL. The MIC value was defined as the lowest concentration of YB allowing no visible growth, and the sub-MIC was defined as the highest concentration of YB that did not inhibit growth by measuring cell density. For other experiments, *S. aureus* was cultured in a 20-mL conical flask with shaking at 37 ^o^C in LB broth containing appropriate concentrations of YB. Bacterial cultures were sampled at intervals of 1 h. Cell density was determined by measuring absorbance at 600 nm.

### RNA extraction

*S. aureus* carrying genes *tsst*-1, *coa*, *sarA*, *icaA*, *cidA*, *sea*, *seb*, *sec*, *sed*, and *see*, were cultured by experimental group with sub-MIC value of the aqueous extract of YB and control group without aqueous extract of YB, respectively. These bacteria were collected at their exponential growth phase. Total bacterial mRNA was isolated using Kit RNA_fast200_ (TaKaRa Biotechnology, China). The mRNA was qualified using ND-2000 ultra-micro nucleic acid protein analyzer (Nanodrop, USA), then was stored at -80 ^o^C before it was used.

### Confocal Laser Scanning Microscope

The 1.5×10^8^ CFU/mL pALC1742 and pALC1743 were prepared. The active ingredients of YB were diluted with TSB broth and configuration of bacteria fluid. Finally, the concentration of YB active ingredient and bacterial fluid were sub-MIC and 5×10^5^ CFU/mL. The biofilm of pALC1740, pALC1742 and pALC1743 were cultivated in laser confocal culture dishes (Shanghai Jingan Biotechnology Co. LTD) at 37 ^o^C for 7 days. The inhibitory effects of YB at different concentrations on *hla expression,* RNA II and RNA III expression, and the effects on the growth of biofilms were observed by a laser confocal scanning microscope TCSSP5II (Leica, Germany) and a fluorescence microscope TE2000-U (Nikon) from the second day. Each reported strain was cultured with TSB broth without YB as the control. Each experiment was repeated three times.

### Rabbit blood plate test

The rabbit blood plate with sub-MIC YB and the normal rabbit blood plate were prepared. 10 μL 1.5×10^8^ CFU/ml ATCC29213 was added to the rabbit blood plate with sub-MIC YB and the normal rabbit blood plate then observed the hemolytic ring after 24 hours.

### Slide and tube coagulase tests

According to the reported method 14, 15, the effects of YB with sub-MIC on bound coagulases were carried out using slide coagulase tests ATCC29213 was cultured in the rabbit blood plate with YB. A drop of EDTA anticoagulant rabbit plasma and some bacterial colony were mixed in clean glass slide, and ATCC29213 with normal rabbit blood plate was cultured as control, both of which were observed within 10 s. The effects of YB with sub-MIC on free coagulases were carried out using tube coagulase tests. ATCC29213 was cultured in the rabbit blood plate with sub-MIC YB. A 1/4 of fresh rabbit plasma 1 ml and six colonies were mixed in 2 mL EP tubes, and ATCC29213 with normal rabbit blood plate was cultured as control, both of which were in water bath at 37 ^o^C for 3 h before they were observed.

### Reverse Transcription-Polymerase Chain Reaction (RT-PCR)

According to the gene sequence in Genebank, using the oligo7 software design RT-PCR primers of reference (16SrRNA) and *cao, sarA*, *icaA*, *cidA*, *tsst*-1,* sea*, *seb*, *sec*, *sed*, *see*, and *seg* gene. The mRNA relative expressions of genes were detected using RT-PCR (ROCHE, LightCycler480 II). The primers were synthesized by Shanghai Jingan Biotechnology Co. LTD. The kits for RT-PCR and kits RNA_fast200_ (RR820A) were purchased from TaKaRa Biotechnology, China. The RT-PCR primers shown in Table 2.

### Statistical analysis

Statistical analyses were performed as previously described using SPSS 20 statistical software 16-18. Measured data are expressed as mean ± standard deviation and analyzed using the *t*-test, χ^2^-test and variations considered significant at *p* < 0.05.

## Results

### Characteristics of the subjects included in the study

Among all prospectively enrolled subjects, 45 cases were stage III HAPU patients, 15 cases were stage IV HAPU patients. The ulcer site of 32 patients was sacrococcygeal region (SR), 17 patients was ischial tuberosity (IT) and 11 patients was greater trochanter (GT). Bacterial culture of wound pathogens in all patients showed *S. aureus* positive. The demographic and clinical characteristics for YB treatment and control subjects were shown in Table 1. No significant difference in terms of age, gender, stage and course of disease were noted between YB treatment and control subjects.

### YB treatment reduces HAPU

To assess the role of YB in the treatment of HAPU patients, 60 cases of HAPU patients were divided into two groups, YB treatment and control group. After 20 days of YB treatment, our results showed that YB treatment can effectively reduce the acne wound area (p < 0.05) and improve efficacy (p < 0.05), seen in Table 3. These results showed that YB treatment can effectively reduce HAPU.

### Antibacterial effect of YB

**Figure 1:**
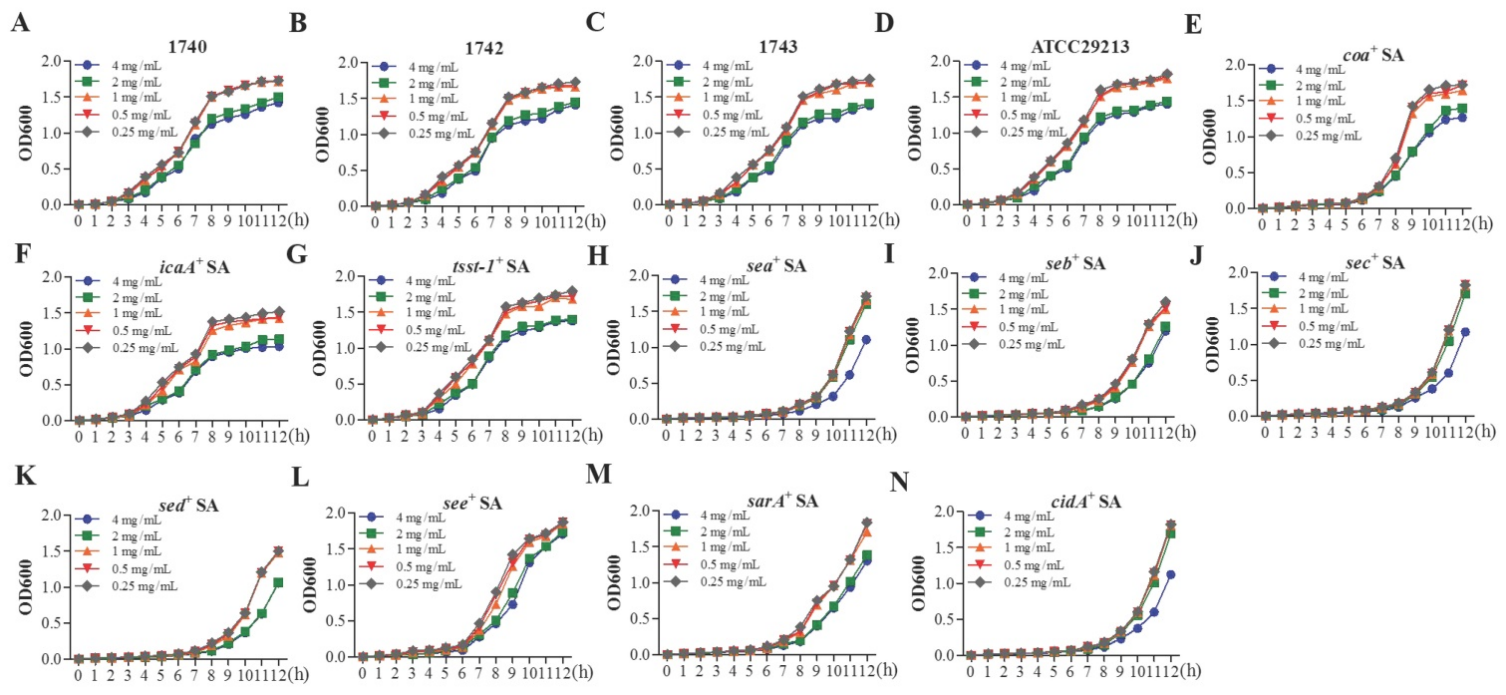
** The role of YB on the growth of *S. aureus.**** S*. *aureus* strain pALC1740 (A), pALC1742 (B), pALC1743 (C), ATCC29213 (D),* coa*^+^ *S*. *aureus* (E), *icaA*^+^ *S*. *aureus* (F), *sarA*^+^ *S*. *aureus* (G), *cidA*^+^ *S*. *aureus* (H), *sea*^+^ *S*. *aureus* (I), *seb*^+^ *S*. *aureus* (J), *sec*^+^ *S*. *aureus* (K), *sed*^+^ *S*. *aureus* (L), *see*^+^ *S*. *aureus* (M) and *tsst-1*^+^ *S*. *aureus* (N) were cultured for 12 h with different concentrations of YB. The effect of YB on the growth of SA was evaluated by measuring OD600.

Above results showed YB treatment reduce acne wound area of HAPU patients with *S. aureus* positive in wound pathogens detecting (Table 1). At present, a number of research reports that* S. aureus* associated with pressure ulcers 19, 20. Therefore, the antibacterial effects of YB on S. aureus were evaluated. First**,** the MIC and sub-MIC values of YB for *S. aureus* pALC1743, pALC1742, pALC1740, and ATCC29213 were 16 mg/mL and 1 mg/mL, respectively. While the MIC value of YB for* S. aureus* carrying genes *coa*, *sarA*, *icaA*, *cidA*, *sea*, *seb*, sec, *sed*, *see*, and* tsst*-1 ranged from 16 mg/mL to 32 mg/mL, and its sub-MIC value ranged from 1 mg/mL to 2 mg/mL. The effects of different concentrations of YB on the growth of pALC1740, pALC1742, pALC1743, ATCC29213,* coa*^+^ *S*. *aureus*, *icaA*^+^ *S*. *aureus*, *sarA*^+^ *S*. *aureus*, *cidA*^+^ *S*. *aureus*, *sea*^+^ *S*. *aureus*, *seb*^+^ *S*. *aureus*, *sec*^+^ *S*. *aureus*, *sed*^+^ *S*. *aureus*, *see*^+^ *S*. *aureus* and *tsst-1*^+^ *S. aureus* were shown in Figure 1.

### YB inhibits HIa expression in *S*. *aureus*

**Figure 2:**
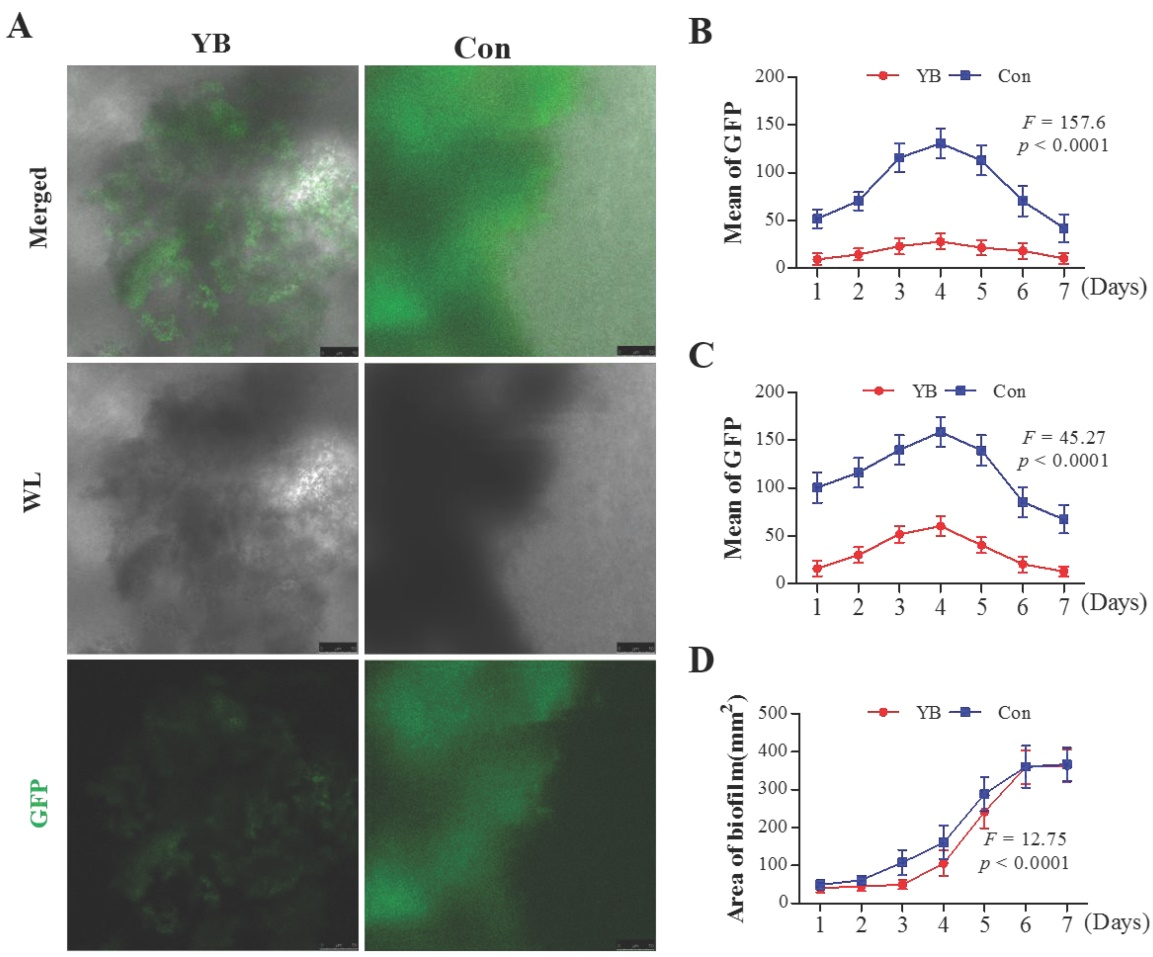
** The role of YB on HIa expression of *S. aureus* strain pALC1740*.***GFP fluorescence intensity was used to indicate HIa expression in YB-treated pALC1740. (A) *hla* promoter activity was evaluated by GFP-mediated fluorescence in YB-treated pALC1740. (B) Overall fluorescence intensity in YB-treated pALC1740. (C) Fluorescence intensity of the largest biofilm in YB-treated pALC1740. (D) The area of the largest biofilm in YB-treated pALC1740. WL: White light.

**α**-hemolysin (Hla) toxin is the most emphasized and characterized virulence factor in *S*. *aureus* 21. Herein, to study the role of YB on virulence gene Hla expression, a standard *S*. *aureus* strain pALC1740, which *hla* promoter fused to a gfp_uvr_ reporter gene was used. Results showed that the GFP-mediated fluorescence attributable to the* hla* promoter activity was lower in YB-treated pALC1740 than in the parental un-treated strain (Figure 2A). GFP fluorescence intensity, used to indicate HIa expression was increased in pALC1740 strain after culturing for 4 days to reach a top, and then decreased after culturing for 7 days (Figure 2B, 2C). However, after YB treatment, overall fluorescence intensity (Figure 2B) and fluorescence intensity of the largest biofilm (Figure 2C) were significantly lower than that in the parental un-treated strain. Additionally, the area of the largest biofilm in YB-treated pALC1740 strain was significantly smaller than that in un-treated strain (Figure 2D). These results suggested YB inhibited HIa expression.

### YB inhibits *agr* system in *S*. *aureus*

**Figure 3:**
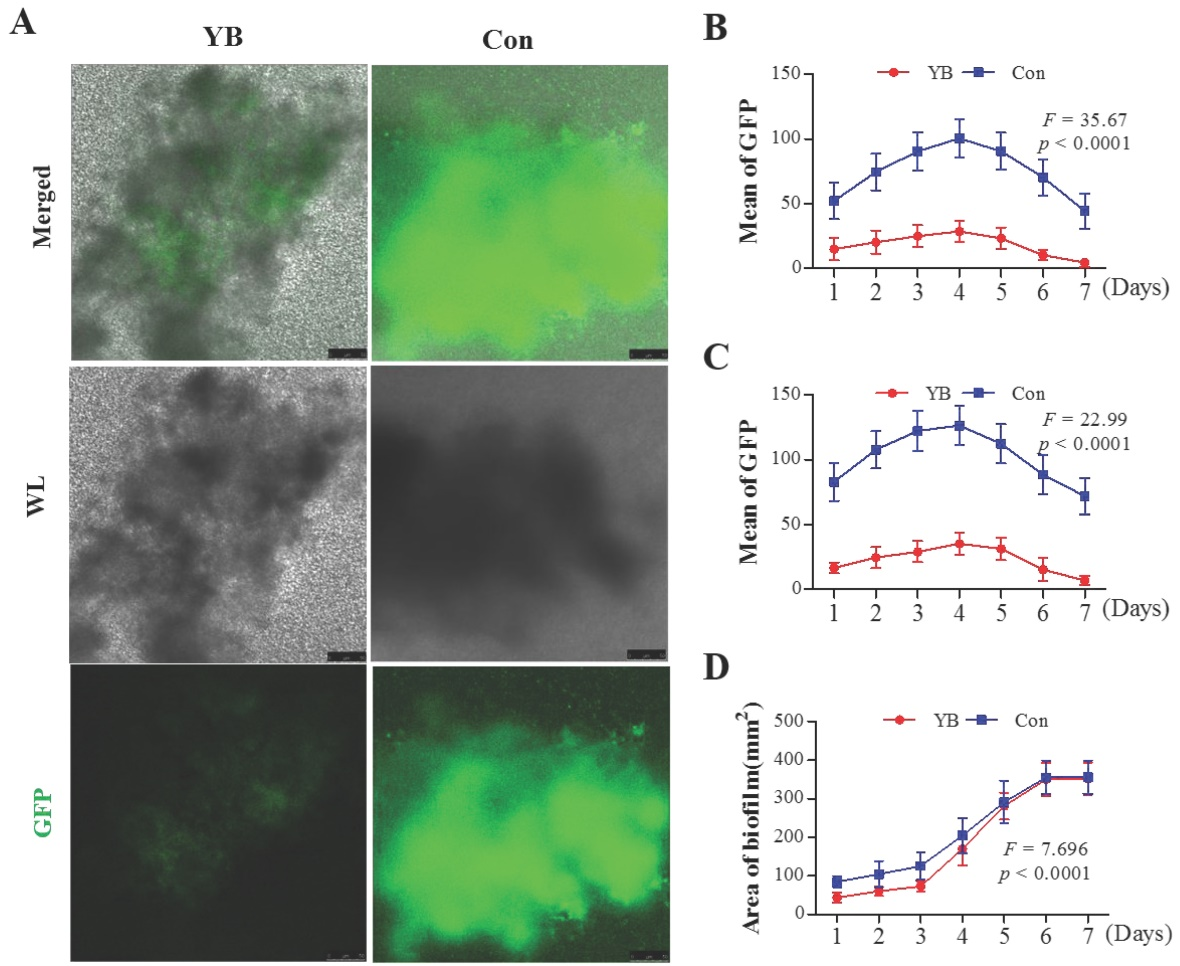
** The role of YB on *agr* system of *S. aureus* strain pALC1742*.***GFP fluorescence intensity was used to indicate *agr* system RNAII or RNAIII promoter in YB-treated pALC1742. (A) RNAII or RNAIII promoter activity was evaluated by GFP-mediated fluorescence in YB-treated pALC1742. (B) Overall fluorescence intensity in YB-treated pALC1742. (C) Fluorescence intensity of the largest biofilm in YB-treated pALC1742. (D) The area of the largest biofilm in YB-treated pALC1742. WL: White light.

**Figure 4:**
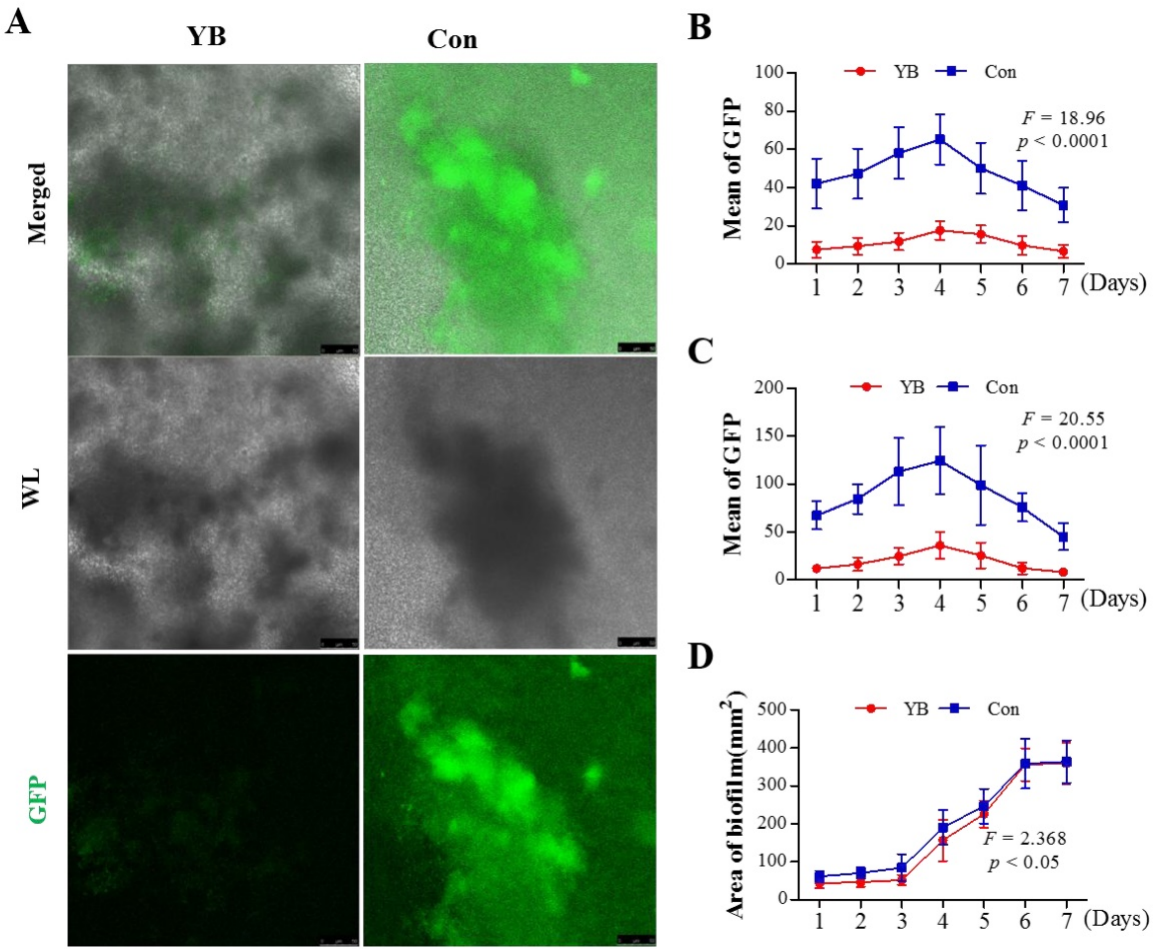
** The role of YB on *agr* system of *S. aureus* strain pALC1743*.***GFP fluorescence intensity was used to indicate *agr* system RNAII or RNAIII promoter in YB-treated pALC1743. (A) RNAII or RNAIII promoter activity was evaluated by GFP-mediated fluorescence in YB-treated pALC1743. (B) Overall fluorescence intensity in YB-treated pALC1743. (C) Fluorescence intensity of the largest biofilm in YB-treated pALC1743. (D) The area of the largest biofilm in YB-treated pALC1743. WL: White light.

Recent studies have shown that most of the virulence factors in *S*. *aureus* are regulated by accessory gene regulator (*agr*) system 22, which comprises two divergent transcripts, RNAII and RNAIII 23, 24. Herein, we want to detect whether the RNAII and RNAIII expression is reduced in YB-treated *S*. *aureus*. To verify this possibility, we also used two standard *S*. *aureus* strain pALC1742 or pALC1743, containing an RNAII or RNAIII promoter linked to gfp_uvr_ reporter gene, respectively. Results showed that the GFP fluorescence intensity and the area of the largest biofilm both on YB-treated pALC1742 strain (Figure 3) and pALC1743 strains (Figure 4) were decreased. These results suggested YB inhibited RNAII and RNAIII expression*.*

### YB inhibits coagulase activity in *S*. *aureus*

**Figure 5:**
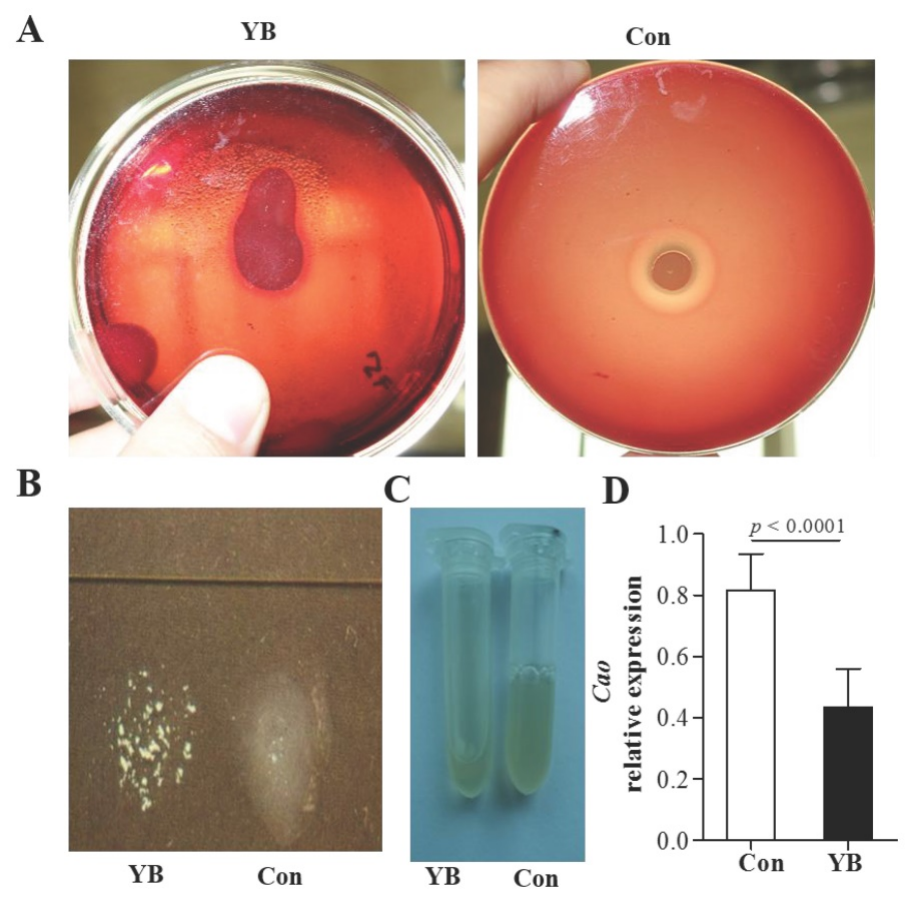
** The role of YB on coagulase activity* of S. aureus* strain ATCC29213*.***ATCC29213 strain was used to detect coagulase activity in *S*. *aureus* by slide coagulase test, tube coagulase test and RT-PCR. (A) Blood plates.(B) Slide coagulase test. (C) Tube coagulase test. (D) The relative expression of *cao* gene in YB-treated ATCC29213 strain was detected by RT-PCR.

ATCC29213 strain was used to detect coagulase activity in *S*. *aureus* by slide coagulase test and tube coagulase test. Results showed that ATCC29213 strain was negative in YB-treated blood plates (Figure 5A).Both slide coagulase test (Figure 5B) and tube coagulase test (Figure 5C) showed ATCC29213 strain has low coagulase activity after YB treatment. Moreover, the relative expression of *cao* gene in YB-treated ATCC29213 strain was significantly lowers than that in control group (Figure 5D). These results suggested YB inhibited coagulase activity.

### YB inhibits enterotoxin and tsst-1

**Figure 6:**
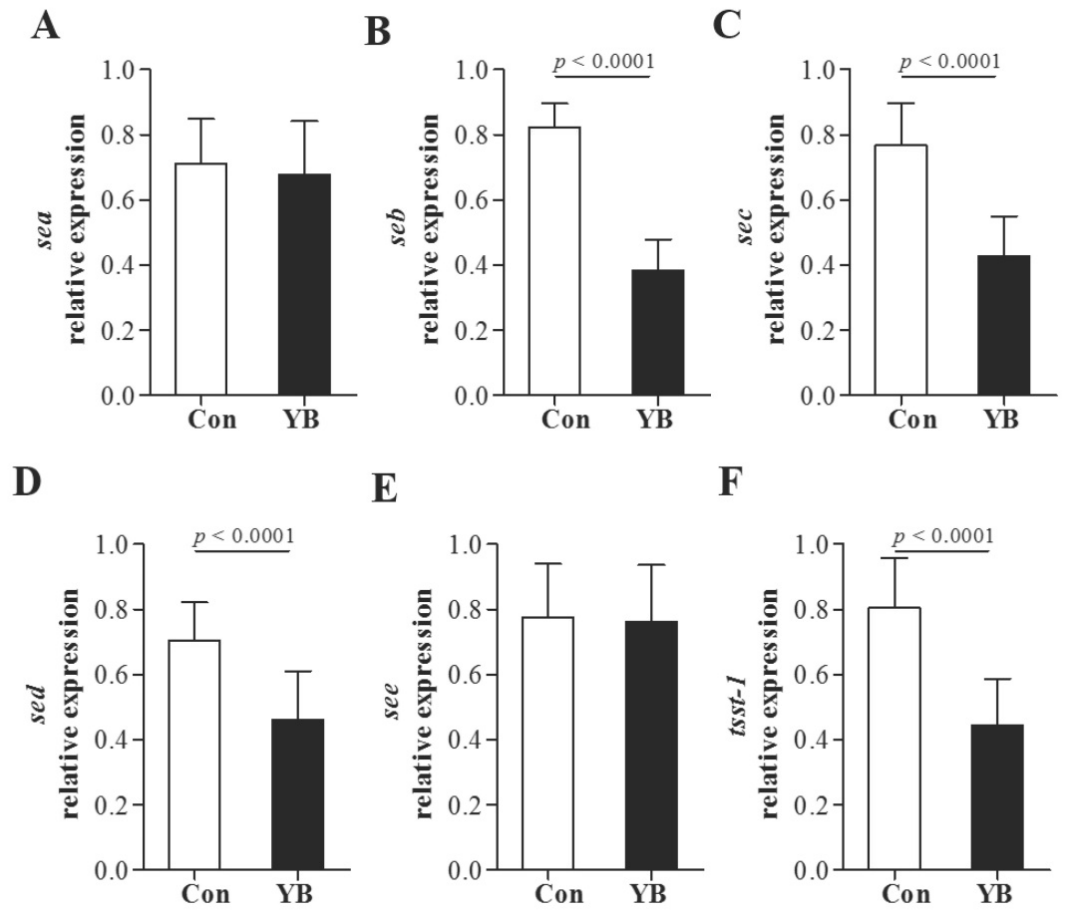
** The role of YB on enterotoxin and tsst-1 expression of* S. aureus.***To further evaluate the role of YB on enterotoxin and tsst-1 secretion, *sea* (A), *seb* (B), *sec* (C), *sed* (D), *see* (E), and *tsst*-*1* (F) positive *S. aureus* were used to enterotoxin and tsst-1 expression using RT-PCR.

To further evaluate the role of YB on enterotoxin and tsst-1 secretion, *sea*, *seb*, *sec*, *sed*, *see*, and *tsst*-1 positive SA were used to enterotoxin and tsst-1 expression using RT-PCR. Results showed that the mRNA expression of *seb*, *sec*, *sed*, and *tsst*-1 were significantly reduced after YB treatment (Figure 6). These results suggested YB inhibited enterotoxin and tsst-1 secretion.

### YB inhibits adhesion function related genes expression

**Figure 7:**
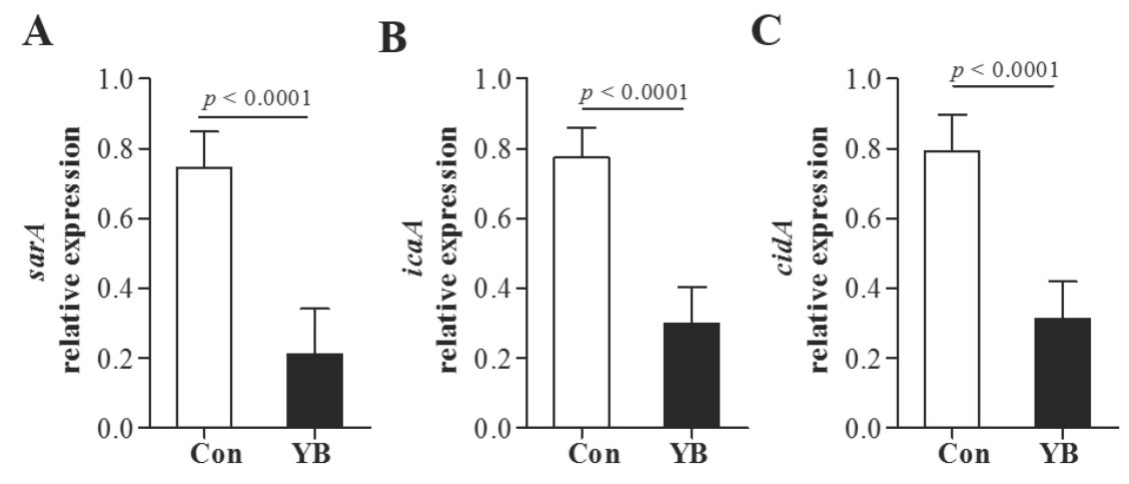
** The role of YB on adhesion function related genes expression of* S. aureus.***YB inhibits adhesion function related genes* sarA* (A), *icaA* (B), and* cidA* (C) mRNA expression in *icaA*, *sarA*, *cidA* positive *S. aureus*.

In addition, YB inhibits adhesion function related genes* sarA*, *icaA*, and* cidA* mRNA expression in *icaA*^+^ *S*. *aureus*, *sarA*^+^ *S*. *aureus*, *cidA*^+^ *S*. *aureus*, respectively (Figure 7).

## Discussion

YB is a secret herbal medicinal formula developed in 1902 by Qu Huangzhang and widely used by Traditional Chinese Medicine (TCM) practitioners to stop bleeding caused by traumatic injury and surgery, haemoptysis, hematochezia, hemorrhoid haemorrhage, metrorrhagia, metrostaxis and ulcer (ulcerative colitis, peptic ulcer, oral ulcer and skin ulcer) in China. Herein, we found that YB treatment can effectively reduce the acne wound area and improve efficacy on HAPU patients with *S. aureus* positive of acne wound pathogens. Further in-depth research showed that the sub-MIC of YB has an inhibitory effects on the expression of *S. aureus agr* system, which may be related to YB containing complex medical plant ingredients. These ingredients can act directly or indirectly on bioactive molecules of the agr system through complex mechanisms, and different from some sub-MICs of antibiotics (such as oxacillin, etc.), which can enhance the expression of *agr* system 25. In view of the fact that sub-MIC is unavoidable in the course of antibiotic treatment, YB can be used as an adjunct therapy for antibiotics and is of great promise in reducing the side effects of antibiotics on HAPU patients.

Recent studies have shown that most of the virulence factors in *S. aureus* are regulated by accessory gene regulator (*agr*) system, which contains two RNA transcription units RNA II and RNA III 22. The transcription of RNA II and RNA III is controlled by the transcriptional promoters P2 and P3. When the synthesis of RNA II and RNA III increases, the secretion of virulence factors from *S. aureus* increases 22, 26. The virulence factors of *S*. *aureus* mainly include α-hemolysin (regulated by *hla*), coagulase (regulated by *cao*), enterotoxin and toxic shock syndrome toxin-1 (*tsst*-1) and so on 27-29. The virulence factor is closely related to the pathogenicity of *S. aureus*, because in most cases, virulence is a pathogenic prerequisite for bacteria, but it is not necessary for bacterial growth. The reason for microbial infection is that the site of infection is coordinated by a certain number of pathogenic bacteria 30, 31. That is to say, as long as the virulence of the bacteria is completely inhibited, even if the bacteria survive, it will not cause the occurrence of infectious diseases. Therefore, the therapeutic regimen of inhibiting virulence not only achieves the purpose of treating the infection but also does not destroy the integrity of the original host flora. Due to the imbalance of bacteria, a series of changes in host immune function and infection can be avoided, which greatly reduces the pressure of antibiotic selection and reduces the probability of occurrence of drug-resistant bacteria 32, 33. Herein, our results showed that the active ingredients of sub-MIC of YB significantly inhibited *α*-hemolysin, coagulase, enterotoxin B, enterotoxin C, enterotoxin D and *tsst*-1.

Possible contributions to these results are as follows: (1) α-hemolysin, coagulase and some enterotoxins regulated by the *agr* system 34. When the sub-MIC of YB significantly inhibited the expression of RNAII and RNAIII, the expression of the relevant virulence factors regulated by the* agr* system was significantly decreased. (2) YB is composed of complex molecules derived from medicinal plants and it can reasonably be concluded that its mechanism or mechanism of action is also complex. These molecules may directly affect the corresponding virulence factors to reduce the production of the related virulence factors. Currently, it has been clearly demonstrated that *agr*-encoded proteins cannot explain all steps in the core autoinduction circuit 35. There was no difference in *sea* and *see* mRNA expression between the experimental and control groups, as the expression of* sea* and *see* mRNA was not regulated by the* agr* system 36. The above analysis shows that the regulatory mechanisms of the virulence factors such as *S. aureus* toxins are complex, and more efforts should be made to deal with the increasingly severe *S. aureus* infection.

Biofilm is closely related to the pathogenicity of *S. aureus* 37. Our research results showed that YB with sub-MIC could significantly inhibited *sarA*, *icaA* and *cidA* mRNA expression. And the area of the largest area of the biofilm of pALC1740, pALC1742 and pALC1743 in the experimental group was significantly smaller than those in the control group when it was cultured for five days. This further confirms that YB can inhibit the formation of biofilms of *S. aureus*. At the initial stage of bacterial adhesion and aggregation, α-hemolysin is required for cell-to-cell interactions during biofilm formation, and the *hla* mutant is unable to fully colonize plastic surfaces under both static and flow conditions 38. Therefore, the sub-MIC of YB could obviously inhibit the biofilm formation when it could obviously inhibit the gene *hla*. The biofilm formed in the observation group was obviously loose than that in the control group, probably because of sub-MIC of YB activity significantly inhibited extracellular DNA (eDNA) regulatory gene *cidA*. eDNA plays an important role in the initial stage of biofilm formation, and is the basis for the construction of mature biofilm 39.

It is worth noting that, in general, inhibition of the *agr* system affects the formation of *S. aureus* biofilm. These results suggest that YB affects *S. aureus* biofilm formation through multiple pathways. To sum up, YB may significantly reduce HAPU via suppressing virulence gene expression and biofilm formation of *S. aureus.*

Herbal therapies exert their therapeutic benefit via various mechanisms, including immune regulation, anti-oxidant activity, inhibition of leukotriene B4 and NF-κB, and antiplatelet activity 40. HAPU is a dysregulated chronic inflammation and may be associated with *S. aureus* infection*.* Herein, we found the Chinese herbal medicine YB may be a potential antimicrobial agent with promising pharmaceutical prospect in resisting *S. aureus* infection on HAPU patients.

## Figures and Tables

**Table 1:** Demographics of subjects included in the two groups of HAPU patients

Group	YB treatment	Control
n	30	30
Age, years	67.15±11.24	64.26±9.58
Male/female, n	18/12	20/10
Stage III/IV, n	21/9	24/6
Ulcer site (SR/IT/GT)	15/10/5	17/7/6
Course of disease, month	18.19±4.26	17.65±3.01
Wound pathogens, n	*SA, 30*	SA, 30

SR: sacrococcygeal region; IT: ischial tuberosity; GT: greater trochanter; SA:* Staphylococcus aureus*

**Table 2:** Primer sequences and source of RT-PCR.

gene	Primer (5′-3′)	GenBank No	Product size (bp)
16SrRNA	16S rRNA-F:GCTGCCCTTTGTATTGTC	CP012692.1	179
	16S rRNA-R:AGATGTTGGGTTAAGTCCC		
*coa*	coa-F:AAAGTTGGAAACCAGCAAGAGG	AB436977.1	98
	coa-R:GTGCCCTGTGGAATTTTAACTAATG		
*sarA*	sarA-F: TGTTTGCTTCAGTGATTCGTTTA	LT671859.1	168
	sarA-R:AACCACAAGTTGTTAAAGCAGTTA		
*icaA*	icaA-F:TGGGATACTGACATGATTACTGAGG	KF972125.1	109
	icaA-R:CAGGCACTAACATCCAGCATAGAG		
*cidA*	cidA-F: ATTCATAAGCGTCTACACCTT	LT671859.1	178
	cidA-R:TTCTTCATACCGTCAGTTGT		
*sea*	sea-F:TTGGAAACGGTTAAAACGAA	LC032460.1	121
	sea-R:GAACCTTCCCATCAAAAACA		
*seb*	seb-F:TGTTCGGGTATTTGAAGATGG	CP013182.1	154
	seb-R:CGTTTCATAAGGCGAGTTGTT		
*sec*	sec-F:GACATAAAAGCTAGGAATTT	CP013955.1	257
	sec-R:AAATCGGATTAACATTATCC		
*sed*	sed-F:CCGTACAAGAATTAGATGC	CP007455.1	166
	sed-R:GGAAAATCACCCTTAACAT		
*see*	see-F:TAATAACCGATTGACCGAAG	M21319.1	277
	see-R:ATCTGGATATTGCCCTTGAG		
*tsst-1*	tst-F:ACCCCTGTTCCCTTATCATC	CP001996.1	108
	tst-R:AAAAGCGTCAGACCCACTAC		

**Table 3:** Treatment effect of YB on HAPU patients

Group	YB treatment	Control	t/χ^2^/HC, p
Acne wound area, cm^2^			
Pre-treatment	28.34±4.24	26.95±3.37	1.41, < 0.05
Post-treatment	2.34±1.24	6.95±1.76	16.82, < 0.05
Secretion disappears, n(%)	26(86.67)	18(60.00)	5.45, < 0.05
Efficacy, n(%)			
Effective	13(43.33)	8(26.67)	2.41, < 0.05
Markedly effective	14(46.67)	9(30.00)	
Improvement	3(10.00)	12(40.00)	
Ineffective	0	1(3.33)	
